# Equivalent Conditions of Generalized Convex Fuzzy Mappings

**DOI:** 10.1155/2014/412534

**Published:** 2014-01-08

**Authors:** Xue Wen Liu, Dou He

**Affiliations:** College of Mathematics, Chongqing Normal University, Chongqing 400047, China

## Abstract

We obtain some equivalent conditions of (strictly) pseudoconvex and quasiconvex fuzzy mappings. These results will be useful to present some characterizations of solutions for fuzzy mathematical programming.

## 1. Introduction

The occurrence of randomness and fuzziness in the real world is inevitable owing to some unexpected situations. In [1], Zadeh initially introduced the concept of fuzzy number. Since then, theories of fuzzy number and their applications have been extensively and intensively studied by many scholars; one can refer to [2–9]. Mathematical programming under fuzzy environment or which involves fuzziness is called fuzzy mathematical programming. Bellman and Zadeh [10] introduced fuzzy optimization problems and stated that a fuzzy decision can be viewed as the intersection of fuzzy goals and problem constraints.

Nanda and Kar [11] proposed the concept of convex fuzzy mappings and proved that a fuzzy mapping is convex if and only if its epigraph is a convex set. At the same time, some applications to fuzzy mathematical programming problems were studied. The convexity has been playing an important role in fuzzy mathematical programming theory. Some related research work has been carried out; one can refer to [12–21]. But it is obvious that the condition of convex fuzzy mappings is too strict. Recently, different types of generalized convex fuzzy mappings were defined. Some properties and the applications were studied in fuzzy mathematical programming problems. Especially, Panigrahi et al. [22] proposed the concept of quasiconvex fuzzy mappings, which is different from that of Nanda and Kar [11] as well as Syau [14], and derived the Karush-Kuhn-Tucker optimality conditions for the constrained fuzzy minimization problems. Strict inequality relation between fuzzy numbers is used in [22], which is too much restrictive. Syau [15] introduced the concept of generalized convexity such as pseudoconvexity for fuzzy mappings with several variables and studied some basic differentiability properties of fuzzy mappings from the standpoint of convex analysis.

Motivated by the earlier works of Panigrahi et al. [22], Karamardian [23], and Karamardian and Schaible [24], in this paper, we establish some equivalent conditions of (strictly) pseudoconvex and quasiconvex fuzzy mappings.

## 2. Preliminaries

In this section, we quote some preliminary notations and definitions.

Let ℝ be the set of all real numbers. A fuzzy number is a mapping *μ* : ℝ → [0,1] with the following properties:

1. *μ*  is normal; that is, [*μ*]_1_ = {*x* ∈ ℝ : *μ*(*x*) = 1} ≠ *∅*,
2. *μ* is upper semicontinuous,
3. *μ* is convex; that is, *μ*(*λx* + (1 − *λ*)*y*) ≥ min{*μ*(*x*), *μ*(*y*)} for all *x*, *y* ∈ ℝ, *λ* ∈ [0,1],
4. the support of *μ*, supp(*μ*) = {*x* ∈ ℝ : *μ*(*x*) > 0} and its closure cl(supp*μ*) is compact.

Let *ℱ* be the set of all fuzzy numbers on ℝ. The *α*-level set of a fuzzy number *μ* ∈ *ℱ*, 0 ≤ *α* ≤ 1, denoted by [*μ*]_*α*_, is defined as

(1) $$\begin{array}{c} {\left[\mu \right]}_{\alpha }=\{\begin{array}{cc} \left\{x\in \mathbb{R}:\mu \left(x\right)\geq \alpha \right\}, & 0<\alpha \leq 1,\\ \mathrm{cl}\left(\mathrm{supp}\left(\mu \right)\right), & \alpha =0. \end{array} \end{array}$$

It is clear that the *α*-level set of a fuzzy number is a closed and bounded interval [*μ*_∗_(*α*), μ*(*α*)]. *μ*_∗_(*α*) denotes the left-hand end point of [*μ*]_*α*_ and μ*(*α*) denotes the right-hand end point of [*μ*]_*α*_. Also any *m* ∈ ℝ can be regarded as a fuzzy number $\tilde{m}$ defined by

(2) $$\begin{array}{c} \tilde{m}\left(t\right)=\{\begin{array}{cc} 1, & t=m,\\ 0, & t\neq m. \end{array} \end{array}$$

In particular, the fuzzy number $\tilde{0}$ is defined as $\tilde{0}(t)=1$ if *t* = 0 and otherwise $\tilde{0}(t)=0$. Thus, fuzzy number *μ* can be identified by parameterized triples {(*μ*_∗_(*α*), μ*(*α*), *α*) : *α* ∈ [0,1]}.

For fuzzy numbers *μ* and *υ* parameterized by

(3) $$\begin{array}{c} \left\{\left(\mu_{*}\left(\alpha \right),\mu^{*}\left(\alpha \right),\alpha \right):\alpha \in \left[0,1\right]\right\},\\ \left\{\left(\upsilon_{*}\left(\alpha \right),\upsilon^{*}\left(\alpha \right),\alpha \right):\alpha \in \left[0,1\right]\right\}, \end{array}$$

respectively, and each nonnegative real number *k*, we define the addition $\mu \;\tilde{+}\;\upsilon$ and nonnegative scalar multiplication *kμ* as follows:

(4) $$\begin{array}{c} \mu \;\tilde{+}\;\upsilon =\left\{\left(\mu_{*}\left(\alpha \right)+\upsilon_{*}\left(\alpha \right),\mu^{*}\left(\alpha \right)+\upsilon^{*}\left(\alpha \right),\alpha \right):\alpha \in \left[0,1\right]\right\},\\ k\mu =\left\{\left(k\mu_{*}\left(\alpha \right),k\mu^{*}\left(\alpha \right),\alpha \right):\alpha \in \left[0,1\right]\right\}. \end{array}$$

Obviously, for each real number *r*,

(5) $$\begin{array}{c} \mu \;\tilde{+}\;r=\left\{\left(\mu_{*}\left(\alpha \right)+r,\mu^{*}\left(\alpha \right)+r,\alpha \right):\alpha \in \left[0,1\right]\right\}. \end{array}$$

Moreover, define the opposite of a fuzzy number *μ* to be the fuzzy number −*μ* satisfying (−*μ*)(*x*) = *μ*(−*x*). In other words, if *μ* is represented by the parametric form {(*μ*_∗_(*α*), μ*(*α*), *α*) : 0 ≤ *α* ≤ 1}, then −*μ* is represented by the corresponding parametric form {(−μ*(*α*), −*μ*_∗_(*α*), *α*) : 0 ≤ *α* ≤ 1}. We represent a fuzzy number *μ* as [*μ*_∗_(*α*), μ*(*α*)].

A fuzzy number *μ* = [*μ*_∗_(*α*), μ*(*α*)] is said to be a triangular fuzzy number if *μ*_∗_(1) = μ*(1). Moreover, if *μ*_∗_(*α*) and μ*(*α*) are linear, then we say that  *μ*  is a linear triangular fuzzy number. We denote 〈*μ*_∗_(0); μ*(1); μ*(0)〉.

**Definition 1 (see [22])**

For *u*, *v* ∈ *ℱ*, we say that *u*≼*v* if for each *α* ∈ [0,1], *u*_∗_(*α*) ≤ *v*_∗_(*α*), u*(*α*) ≤ v*(*α*). If *u*≼*v*, *v*≼*u*, then *u* = *v*. We say that *u*≺*v*, if *u*≼*v* and there exists *α*_0_ ∈ [0,1] such that *u*_∗_(*α*_0_) < *v*_∗_(*α*_0_) or u*(*α*_0_) < v*(*α*_0_). For *u*, *v* ∈ *ℱ*, if either *u*≼*v* or *v*≼*u*, then we say that *u* and *v* are comparable, otherwise noncomparable.

A mapping *F* : *K*⊆ℝ^*n*^ → *ℱ* is said to be a fuzzy mapping. For any *α* ∈ [0,1] and for any *x* ∈ *K*, we denote *F*(*x*) = [*F*_∗_(*x*, *α*), F*(*x*, *α*)].

**Definition 2 (see [22])**

Let *F* : *K*⊆ℝ^*n*^ → *ℱ* be a fuzzy mapping. Then, *F* is said to be comparable if for each pair *x* ≠ *y* ∈ *K*, *F*(*x*) and *F*(*y*) are comparable. Otherwise, *F* is said to be noncomparable.

**Definition 3 (see [22])**

Let *K*⊆ℝ^*n*^ be an open set and assume that *F* : *K* → *ℱ* is a fuzzy mapping. Let *x* = (*x*_1_, *x*_2_,…, *x*_*n*_) ∈ *K* and let *D*_*x*_*i*__, *i* = 1,2,…, *n* stand for the partial differentiation with respect to the *i*th variable *x*_*i*_. Assume that for all *α* ∈ [0,1], *F*_∗_(*x*, *α*) and F*(*x*, *α*) have continuous partial derivatives. Define

(6) DxiF(x)[α]=[DxiF∗(x,α),DxiF∗(x,α)],for  i=1,2,…,n,  α∈[0,1].

If for each *i* = 1,2,…, *n*, *D*_*x*_*i*__ *F*(*x*)[*α*] defines the *α*-cut of a fuzzy number, then we say that *F* is differentiable at *x*, and we denote

(7) $$\begin{array}{c} \tilde{\nabla }F\left(x\right)=\left(D_{x_{1}}F\left(x\right),D_{x_{2}}F\left(x\right),\ldots ,D_{x_{n}}F\left(x\right)\right). \end{array}$$

We call $\tilde{\nabla }F(x)$ the gradient of the fuzzy mapping *F* at *x*. A fuzzy mapping *F* is said to be differentiable at *x* if $\tilde{\nabla }F(x)$ exists and both *F*_∗_(*x*, *α*) and F*(*x*, *α*) for each *α* ∈ [0,1] are differentiable at *x*.

**Definition 4 (see [22])**

Let *K*⊆ℝ^*n*^ be a nonempty open convex set and let *F* : *K* → *ℱ* be a differentiable fuzzy mapping. *F* is said to be pseudoconvex if for each *x*, *y* ∈ *K*, $\tilde{0}≼\tilde{\nabla }F{\left(x\right)}^{T}(y-x)$ implies that *F*(*x*)≼*F*(*y*).

**Definition 5 (see [22])**

Let *K*⊆ℝ^*n*^ be a nonempty convex set and let *F* : *K* → *ℱ* be a fuzzy mapping. *F* is said to be quasiconvex if for each *x*, *y* ∈ *K* and for each *λ* ∈ (0,1), the following implications hold:

(8) $$\begin{array}{c} F\left(\left(\lambda x+\left(1-\lambda \right)y\right)\right)≼\max\left\{F\left(x\right),F\left(y\right)\right\}, \end{array}$$

whenever *F*(*x*) and *F*(*y*) are comparable.

**Definition 6 (see [25])**

Let *K*⊆ℝ^*n*^ be a nonempty open convex set and let *F* : *K* → *ℱ* be a differentiable fuzzy mapping. *F* is said to be strictly pseudoconvex if for each *x*, *y* ∈ *K*, $\tilde{0}≼\tilde{\nabla }F{\left(x\right)}^{T}(y-x)$ implies that *F*(*x*)≺*F*(*y*).

In the following sections, we always assume that *K*⊆ℝ^*n*^ be a nonempty open convex set, *F* : *K*⊆ℝ^*n*^ → *ℱ* be a differentiable fuzzy mapping, and *F* be comparable.

## 3. Pseudoconvexity of Fuzzy Mappings

In this section, we establish the equivalent conditions of pseudoconvex and strictly pseudoconvex fuzzy mappings. We first give some lemmas which will be used in the sequel.

**Lemma 7 (see [25])**

Assume that *F* is a pseudoconvex fuzzy mapping. Then *F* is a quasiconvex fuzzy mapping.

**Lemma 8 (see [22])**

*F* is a quasiconvex fuzzy mapping if and only if for each *x*, *y* ∈ *K*, *F*(*x*)≼*F*(*y*) implies that $\tilde{\nabla }F{\left(y\right)}^{T}(x-y)≼\tilde{0}$.

**Theorem 9**

*F* is a pseudoconvex fuzzy mapping if and only if for each *x*, *y* ∈ *K*, $\tilde{0}≼\tilde{\nabla }F{\left(x\right)}^{T}(y-x)$ implies that $\tilde{0}≼\tilde{\nabla }F{\left(y\right)}^{T}(y-x)$.

**Proof**

Suppose that *F* is a pseudoconvex fuzzy mapping. Let *x*, *y* ∈ *K* be such that

(9) $$\begin{array}{c} \tilde{0}≼\tilde{\nabla }F{\left(x\right)}^{T}\left(y-x\right). \end{array}$$

We need to show that $\tilde{0}≼\tilde{\nabla }F{\left(y\right)}^{T}(y-x)$. Assume to the contrary that

(10) $$\begin{array}{c} \tilde{\nabla }F{\left(y\right)}^{T}\left(y-x\right)≺\tilde{0}. \end{array}$$

By the pseudoconvexity of *F* and (9), we have

(11) $$\begin{array}{c} F\left(x\right)≼F\left(y\right). \end{array}$$

By Lemmas 7 and 8, it follows from (11) that

(12) $$\begin{array}{c} \tilde{0}≼\tilde{\nabla }F{\left(y\right)}^{T}\left(y-x\right), \end{array}$$

which contradicts (10).

Conversely, let *x*, *y* ∈ *K* such that

(13) $$\begin{array}{c} \tilde{0}≼\tilde{\nabla }F{\left(x\right)}^{T}\left(y-x\right). \end{array}$$

We need to show that *F*(*x*)≼*F*(*y*). Assume the contrary, that is, *F*(*y*)≺*F*(*x*). Hence, for each *α* ∈ [0,1],

(14) $$\begin{array}{c} F_{*}\left(y,\alpha \right)\leq F_{*}\left(x,\alpha \right),\;\;F^{*}\left(y,\alpha \right)\leq F^{*}\left(x,\alpha \right), \end{array}$$

and there exists an *α*_0_ ∈ [0,1] such that

(15) $$\begin{array}{c} F_{*}\left(y,\alpha_{0}\right)<F_{*}\left(x,\alpha_{0}\right)\;\text{or}\;F^{*}\left(y,\alpha_{0}\right)<F^{*}\left(x,\alpha_{0}\right). \end{array}$$

Without loss of generality, we assume that

(16) $$\begin{array}{c} F_{*}\left(y,\alpha_{0}\right)<F_{*}\left(x,\alpha_{0}\right). \end{array}$$

From the mean-value theorem, we have

(17) $$\begin{array}{c} F_{*}\left(y,\alpha_{0}\right)-F_{*}\left(x,\alpha_{0}\right)=\nabla F_{*}{\left(\bar{x},\alpha_{0}\right)}^{T}\left(y-x\right), \end{array}$$

where $\bar{x}=x+\lambda (y-x)$ for some *λ* ∈ (0,1). From (16) and (17), we have

(18) $$\begin{array}{c} 0<\nabla F_{*}{\left(\bar{x},\alpha_{0}\right)}^{T}\left(x-\bar{x}\right). \end{array}$$

On the other hand, from (13), it follows that

(19) $$\begin{array}{c} \tilde{0}≼\tilde{\nabla }F{\left(x\right)}^{T}\left(\bar{x}-x\right). \end{array}$$

From the comparability assumption of *F*, this implies that

(20) $$\begin{array}{c} \tilde{0}≼\tilde{\nabla }F{\left(\bar{x}\right)}^{T}\left(\bar{x}-x\right). \end{array}$$

Then, for each *α* ∈ [0,1], we have

(21) $$\begin{array}{c} 0\leq \nabla F_{*}{\left(\bar{x},\alpha \right)}^{T}\left(\bar{x}-x\right), \end{array}$$

which contradicts (18).

**Remark 10**

Theorem 9 generalizes Karamardian's result (Theorem  3.1 in [23]) to fuzzy mapping case.

**Theorem 11**

*F* is a strictly pseudoconvex fuzzy mapping if and only if for each *x*, *y* ∈ *K*, *x* ≠ *y*, $\tilde{0}≼\tilde{\nabla }F{\left(x\right)}^{T}(y-x)$ implies that $\tilde{0}≺\tilde{\nabla }F{\left(y\right)}^{T}(y-x)$.

**Proof**

Suppose that *F* is a strictly pseudoconvex fuzzy mapping. Let *x*, *y* ∈ *K*, *x* ≠ *y*, such that

(22) $$\begin{array}{c} \tilde{0}≼\tilde{\nabla }F{\left(x\right)}^{T}\left(y-x\right). \end{array}$$

We need to show that

(23) $$\begin{array}{c} \tilde{0}≺\tilde{\nabla }F{\left(y\right)}^{T}\left(y-x\right). \end{array}$$

Assume to the contrary that

(24) $$\begin{array}{c} \tilde{\nabla }F{\left(y\right)}^{T}\left(y-x\right)≼\tilde{0}. \end{array}$$

Combine with (22) and from strict pseudoconvexity of *F*, we have

(25) $$\begin{array}{c} F\left(x\right)≺F\left(y\right). \end{array}$$

On the other hand, (24) can be written as

(26) $$\begin{array}{c} \tilde{0}≼\tilde{\nabla }F{\left(y\right)}^{T}\left(x-y\right). \end{array}$$

From strict pseudoconvexity of *F*, it follows that

(27) $$\begin{array}{c} F\left(y\right)≺F\left(x\right), \end{array}$$

which contradicts (25).

Conversely, let *x*, *y* ∈ *K*, *x* ≠ *y* such that

(28) $$\begin{array}{c} \tilde{0}≼\tilde{\nabla }F{\left(x\right)}^{T}\left(y-x\right). \end{array}$$

We need to show that *F*(*x*)≺*F*(*y*). Assume to the contrary that

(29) $$\begin{array}{c} F\left(y\right)≼F\left(x\right). \end{array}$$

Hence, for each *α* ∈ [0,1],

(30) $$\begin{array}{c} F_{*}\left(y,\alpha \right)\leq F_{*}\left(x,\alpha \right),\;\;F^{*}\left(y,\alpha \right)\leq F^{*}\left(x,\alpha \right). \end{array}$$

From the mean-value theorem, we have

(31) $$\begin{array}{c} F_{*}\left(y,\alpha \right)-F_{*}\left(x,\alpha \right)=\nabla F_{*}{\left(\bar{x},\alpha \right)}^{T}\left(y-x\right), \end{array}$$

where $\bar{x}=x+\lambda (y-x)$ for some *λ* ∈ (0,1).

From (30) and (31), we have

(32) $$\begin{array}{c} 0\leq \nabla F_{*}{\left(\bar{x},\alpha \right)}^{T}\left(x-\bar{x}\right). \end{array}$$

On the other hand, from (28), it follows that $\tilde{0}≼\tilde{\nabla }F{\left(x\right)}^{T}(\bar{x}-x)$, which implies that $\tilde{0}≺\tilde{\nabla }F{\left(\bar{x}\right)}^{T}(\bar{x}-x)$. Then, for each *α* ∈ [0,1],

(33) $$\begin{array}{c} 0<\nabla F_{*}{\left(\bar{x},\alpha \right)}^{T}\left(\bar{x}-x\right), \end{array}$$

which contradicts (32).

**Remark 12**

Theorem 11 generalizes Karamardian and Schaible's result (Proposition  4.1 in [24]) to fuzzy mapping case.

## 4. Quasiconvexity of Fuzzy Mappings

In this section, we establish an equivalent condition of a differentiable quasiconvex fuzzy mapping.

**Theorem 13**

*F* is a quasiconvex fuzzy mapping if and only if for each *x*, *y* ∈ *K*, $\tilde{0}≺\tilde{\nabla }F{\left(x\right)}^{T}(y-x)$ implies that $\tilde{0}≼\tilde{\nabla }F{\left(y\right)}^{T}(y-x)$.

**Proof**

Suppose that *F* is a quasiconvex fuzzy mapping. Let *x*, *y* ∈ *K* such that

(34) $$\begin{array}{c} \tilde{0}≺\tilde{\nabla }F{\left(x\right)}^{T}\left(y-x\right). \end{array}$$

The relation *F*(*y*)≼*F*(*x*) is not possible. Otherwise, it will imply that

(35) $$\begin{array}{c} \tilde{\nabla }F{\left(x\right)}^{T}\left(y-x\right)≼\tilde{0}, \end{array}$$

according to Lemma 8, which contradicts to (34). From the compatibility,

(36) $$\begin{array}{c} F\left(x\right)≺F\left(y\right). \end{array}$$

From Lemma 8 and (36), it follows that

(37) $$\begin{array}{c} \tilde{\nabla }F{\left(y\right)}^{T}\left(x-y\right)≼\tilde{0}; \end{array}$$

that is,

(38) $$\begin{array}{c} \tilde{0}≼\tilde{\nabla }F{\left(y\right)}^{T}\left(y-x\right). \end{array}$$

Conversely, assume that *F* is not a quasiconvex fuzzy mapping. Then, there exists *x*, *y* ∈ *K*, *λ* ∈ (0,1) such that *F*(*x*)≼*F*(*y*) and $F(y)≼F(x)≺F(\bar{x})$, where $\bar{x}=x+\lambda (y-x)$. There exists *α* ∈ [0,1] such that

(39) $$\begin{array}{c} F_{*}\left(y,\alpha \right)\leq F_{*}\left(x,\alpha \right)<F_{*}\left(\bar{x},\alpha \right), \end{array}$$

or

(40) $$\begin{array}{c} F^{*}\left(y,\alpha \right)\leq F^{*}\left(x,\alpha \right)<F^{*}\left(\bar{x},\alpha \right). \end{array}$$

Without loss of generality, we assume that

(41) $$\begin{array}{c} F_{*}\left(y,\alpha \right)\leq F_{*}\left(x,\alpha \right)<F_{*}\left(\bar{x},\alpha \right). \end{array}$$

By the mean-value theorem, then there exist *z*_1_, *z*_2_ such that

(42) $$\begin{array}{c} F_{*}\left(\bar{x},\alpha \right)-F_{*}\left(x,\alpha \right)=\nabla F_{*}{\left(z_{1},\alpha \right)}^{T}\left(\bar{x}-x\right),\\ F_{*}\left(\bar{x},\alpha \right)-F_{*}\left(y,\alpha \right)=\nabla F_{*}{\left(z_{2},\alpha \right)}^{T}\left(\bar{x}-y\right), \end{array}$$

where

(43) $$\begin{array}{c} z_{1}=x+\lambda_{1}\left(y-x\right),\\ z_{2}=x+\lambda_{2}\left(y-x\right),\;0<\lambda_{1}<\lambda <\lambda_{2}<1. \end{array}$$

From (41), and (42), it follows that

(44) $$\begin{array}{c} 0<\nabla F_{*}{\left(z_{1},\alpha \right)}^{T}\left(\bar{x}-x\right),\\ 0<\nabla F_{*}{\left(z_{2},\alpha \right)}^{T}\left(\bar{x}-y\right). \end{array}$$

Thus, (43) yields

(45) $$\begin{array}{c} 0<\nabla F_{*}{\left(z_{1},\alpha \right)}^{T}\left(z_{2}-z_{1}\right), \end{array}$$

(46) $$\begin{array}{c} 0<\nabla F_{*}{\left(z_{2},\alpha \right)}^{T}\left(z_{1}-z_{2}\right). \end{array}$$

On the other hand, from (45) and the hypothesis, for each *x*, *y* ∈ *K*,

(47) $$\begin{array}{c} \tilde{0}≺\tilde{\nabla }F{\left(x\right)}^{T}\left(y-x\right)\;\text{implies}\;\;\text{that}\;\;\tilde{0}≼\tilde{\nabla }F{\left(y\right)}^{T}\left(y-x\right), \end{array}$$

we obtain that $\tilde{0}≼\tilde{\nabla }F{\left(z_{2}\right)}^{T}(z_{2}-z_{1})$. Hence,

(48) $$\begin{array}{c} 0\leq \nabla F_{*}{\left(z_{2},\alpha \right)}^{T}\left(z_{2}-z_{1}\right), \end{array}$$

which contradicts (46).

**Remark 14**

Theorem 13 generalizes Karamardian and Schaible's result (Proposition  5.2 in [24]) to fuzzy mapping case.
